# Omnidirectional Light Capture by Solar Cells Mimicking the Structures of the Epidermal cells of Leaves

**DOI:** 10.1038/s41598-019-49046-8

**Published:** 2019-09-04

**Authors:** Min Ju Yun, Yeon Hyang Sim, Seung I. Cha, Dong Yoon Lee

**Affiliations:** 1 Energy Conversion Research Center, Electrical Materials Research Division, Korea Electrotechnology Research Institute, Changwon, 51543 Korea; 2 Department of Electro-functionality Materials Engineering, University of Science and Technology, Changwon, South Korea

**Keywords:** Energy harvesting, Solar energy

## Abstract

It is important to develop solar cells that can capture and utilize omnidirectional light in urban environments, where photovoltaic (PV) devices are installed in fixed directions. We report a new design for such light capture, which mimics the structure of a leaf epidermis. First, we analyzed the epidermal structures of different plant species in detail so that we could copy them and fabricate light-trapping layers with different shapes: as lens arrays, pillars, and lens arrays with rough surfaces. Then we analyzed the results of two-dimensional ray-tracing simulations of perfectly aligned and Gaussian-scattered incident light in terms of light-trapping capabilities. Based on these results, we prepared high-performance dye-sensitized solar cells with light-trapping layers that exhibited omnidirectional light capturing functionality. Our layers enhanced the efficiency of obliquely incident light capture by 70%. Therefore, we expect that new possibilities for next-generation PVs, extending beyond the current rigid concepts, will arise upon the application of these results and from findings that build on these results.

## Introduction

The use of solar cells has expanded into building-integrated photovoltaic (BIPV) and electronics-integrated photovoltaic (EIPV) systems, where PV devices are fixed in a particular direction^1,2^. One way to improve such systems would be to develop solar cells with omnidirectional light-capturing capabilities. Interestingly, BIPV and EIPV systems are similar to most plants in the sense that they are fixed to the ground. However, plants have specific structures that are optimized to collect and utilize incoming light for photosynthesis^3–5^. These structures are composed of a wide range of components, including crown aspects, phyllotaxis arrangements, and dedicated parts of the leaf anatomy. If these structures can be copied to a degree, it may be possible to develop PV systems that produce electrical energy more efficiently.

To this end, leaf anatomy, which includes a cuticle layer, upper epidermal structures, palisade cells, and spongy cells^6–8^, could be good source of inspiration. Epidermal structures in particular could be important for omnidirectional light trapping because they are the first part of the leaf encountered by incoming photons^7,9–11^.

Dye-sensitized solar cells (DSSCs) mimick the circulation of light during photosynthesis, which is then converted from solar energy into electrical energy^10,12^. Therefore, developing a light-trapping layer that captures and converts omnidirectional light in a similar manner to plants would be a promising contribution to the application of PV technology in urban settings. However, few efforts have been made to improve the performance of the PV components of DSSCs by introducing omnidirectional light-trapping capabilities. The vast majority of reported results present light distribution management strategies based on the results of systemic or structural-level analyses rather than simple attempts to replicate entire leaf surfaces and incorporate the replicated films into solar cells^13–21^.

In this study, we analyzed various types of light-trapping layers, modeled after the epidermal structures of leaves, for use in DSSCs to capture and utilize omnidirectional incident light (instead of relaying on vertical light only). We investigated the epidermal structures of different plant species in detail so that we could copy them and fabricate light-trapping layers with different shapes: as lens arrays, pillars, and lens arrays with rough surfaces. It would be first try study by mimicking structural epidermal cell which modify the distribution of incident light in leaf structure. Then we analyzed the results of two-dimensional (2D) ray-tracing simulations of perfectly aligned and Gaussian-scattered incident light in terms of light-trapping capabilities. Based on these results, we prepared high-performance dye-sensitized solar cells with light-trapping layers that exhibited omnidirectional light capturing functionality. Our layers enhanced the efficiency of obliquely incident light capture by 70%. To the best of our knowledge, this is the first step towards the systematic application of leaf structures to PVs. Indeed, we emphasize that these gains in efficiency were achieved with a primitive design; there is much room for further improvement. Therefore, researchers developing the next generation of PVs will gain valuable information from more systematic and detailed analyses of the relevant parts of leaf anatomy and the effects of adopting these techniques.

## Results and Discussion

**Figure 1:**
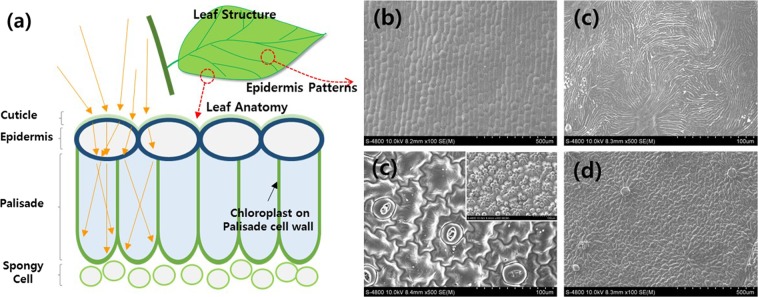
Schematic diagram of the anatomy of a leaf structure. Scanning electron microscopy (SEM) images of the surfaces of leafs from (**b**) *Polygonatum odoratum*, (**c**) *Lagerstroemia indica*, (**d**) *Zinnia elegans* (inset: petal), and (**e**) *Solanum melongena*.

Cells in the epidermis play a role in controlling the distribution of light to palisade cells, where most of the photosynthesis process takes place. Hence, it can be assumed that epidermal cells disperse light in a way such that only the amount of light necessary for photosynthesis is transmitted to the palisade cells, as shown in Fig. 1(a).

The distribution of light within the leaf, and resulting photosynthetic capacity, depends strongly on the geometry of the interface between the cells in the epidermis, which distributes the light, and palisade cells, which absorb it. These cells can be combined in many different ways, and their combinations vary with environmental conditions.

However, the distribution of light is not solely dependent on epidermal cells; considering the process of light passing from the epidermal cells to palisade cell, combination of each consisted cells also plays a role. Nonetheless, incident photons encounter epidermal cells first, and these cells affect the path along which photons travel as they traverse the epidermis and are absorbed into the leaf structure. Therefore, the epidermis plays a core role, and could help explain why it comes in many shapes, depending on the species of plant, crown structure, and external environment. This variation ensures that the distribution of light within the leaf is optimized for photosynthesis.

There are very interesting differences between the epidermal structures of different plant species, and they vary widely depending on the environmental conditions in which they have evolved. For example, the microstructures of surfaces of *Polygonatum odoratum* leaves consist of lines of lens-like arrays (Fig. 1b) whereas *Lagerstroemia indica* has continuous valley-like depressions (Fig. 1c). In addition, there are pillar structures in the leaves of *Zinnia elegans* (Fig. 1d) whereas the leaves of *Solanum melongena* have a mixture of pillar structures and lens arrays (Fig. 1e).

In this study, we tested several epidermal structures, including a lens array, pillar array, and lens array with rough surfaces, which we fabricated with reference to analyzed images of leaf surfaces. Then we confirmed their omni-directional light-capturing behavior and their ability to enhance the performance of DSSCs. We also mimicked the cylindrical morphologies of palisade cells as photoanodes with a 600 µm sized pattern. To use the incoming light transmitted through the light-trapping lens efficiently, it is appropriate to use a patterned photoanode fitted to each light-trapping lens rather than a flat photoanode deposited over the whole area of the electrode. The performances of DSSCs with patterned photoanodes reduce the recombination rate and increase the diffusion rate of incident light^22^. Through light-trapping lens attached on each patterned photoanode, it is expected that light travel path is increased within patterned photoanode and oblique illumination is effectively utilized (Fig. S1).

**Figure 2:**
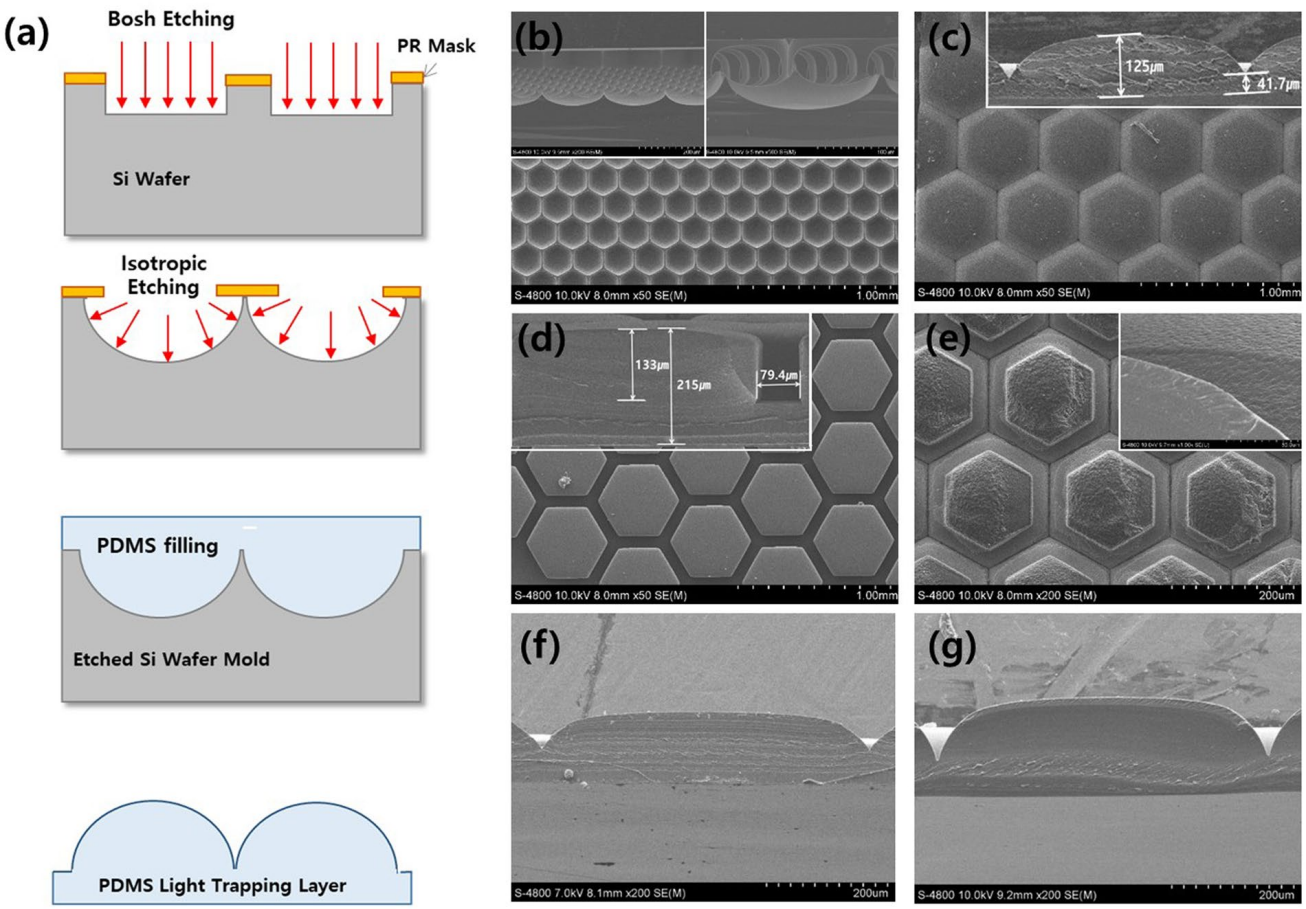
(**a**) Schematic diagram of the fabrication process of light-trapping layers, from etching the silicon (Si) wafer to molding the polydimethysiloxane (PDMS). Scanning electron microscopy (SEM) images of the (**b**) Si wafer master mould in plane and cross-sectional view and light-trapping layers with a (**c**) lens array, (**d**) pillar-shaped lens array, (**e**) lens array with rough surfaces (inset: cross-sections), (**f**) flat-topped lens array, and (**g**) lens array with spaces between patterns.

The light-trapping layers were fabricated by casting polydimethylsiloxane (PDMS) onto patterned silicon (Si) wafers etched through a two-step etching process, as shown in Fig. 2(a). In the first step, we performed straight etching of a Photoresist (PR)-patterned Si wafer to the designed depth. Then the lens shapes were fabricated by isotropic etching. Etched Si wafers were used as master molds for lens arrays, as shown in Fig. 2(b). Then the lens array-based light-trapping layer was cast on top of it, as shown in Fig. 2(c). We fabricated other types of lens structures by controlling the etching depth at each etching step. The pillar structure as shown in Fig. 2(d) was formed by deepened Bosch etching and shortened isotropic etching. And the lens structures with rough surfaces was formed deeper isotropic etching than convex lens as shown in Fig. 2(e). In the case of the pillar structures, hexagonal-shaped pillars of 120 µm height were arranged with 80 µm spacing. Detailed images of the master mold and lens arrays are shown in Fig. S2. The lens arrays with rough surfaces consisted of same as lens arrays, but top of the lens, one more layer was covered with rough surface formed as etching condition (detailed master mold images are shown in Fig. S3) which were expected to reduce Fresnel type reflection in the case of highly oblique incident photons.

To determine the effects of each type of lens, we fabricated two more types of arrays. One had a flat top with rounded edges, as shown in Fig. 2(f). The other had the same shape as the conventional lens array structure but with 10 µm spacing, as shown in Fig. 2(g). Different light-trapping layers may modify the distribution of the incident light in different ways. Hence, this was done to gather data that may help lead to better light-capturing layers that mimic those of epidermal cells.

**Figure 3:**
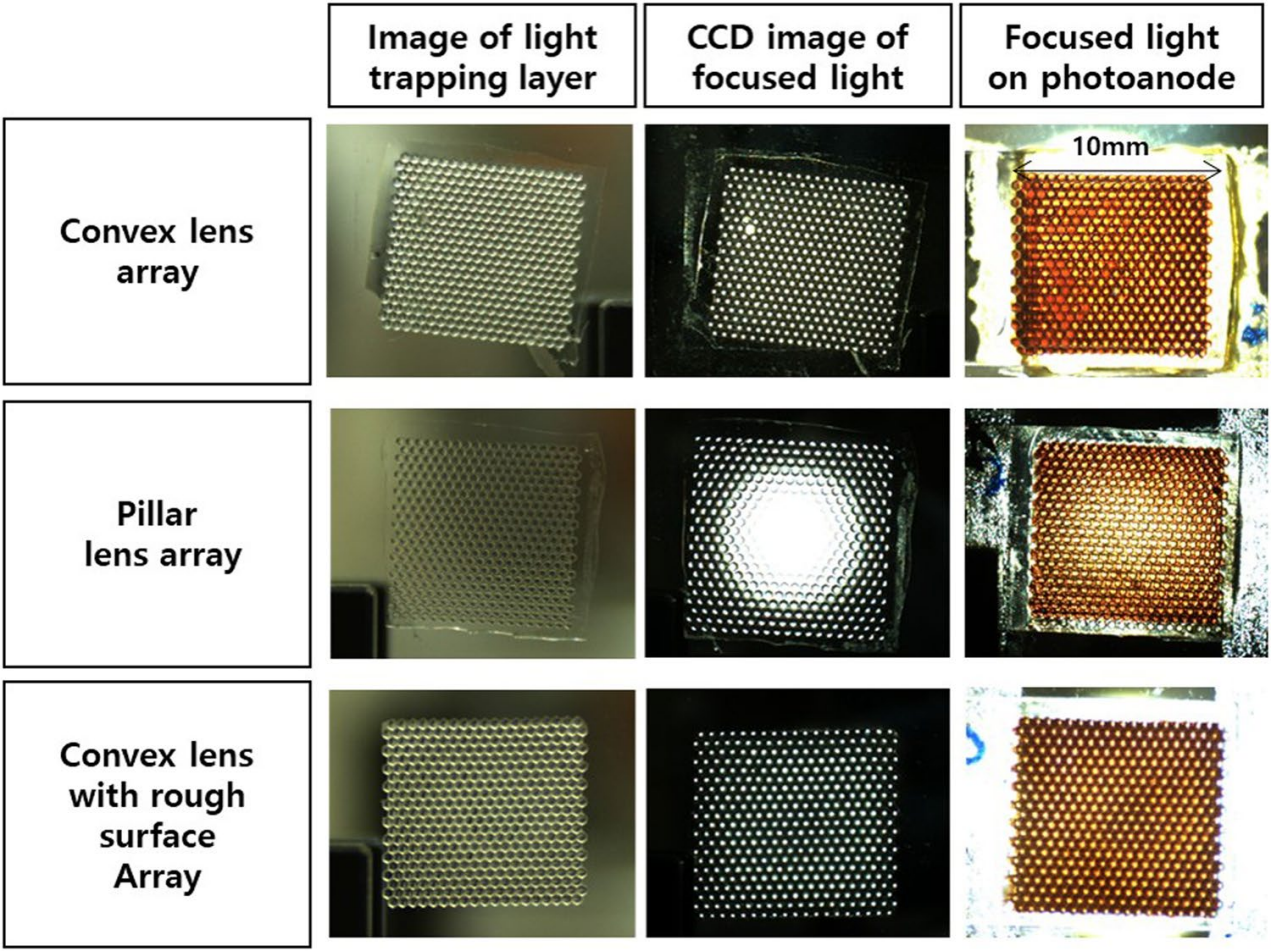
Charge coupled device (CCD) images of light-trapping layers composed of a (**a**) lens array, (**b**) pillar-shaped lens array, and **(c**) lens array with rough surfaces. The images on the left are the light-trapping lenses, those in the middle are the light distribution and focus of each light-trapping layer, and those on the right are photoanodes with light-trapping layers when illuminated by a light-emitting diode source.

We used a charge-coupled device (CCD) to image the light-trapping effects as we illuminated the DSSCs with a light-emitting diode (LED), as shown in Figs 3 and S4. As shown in Fig. 3(a), diffused incoming light focused at the same point was distributed homogeneously into each pattern by the light-trapping layers made of lens arrays with different shapes. This resulted in similar intensities across the whole area. Also we confirmed that light-trapping lenses were correctly raised on each patterned photoanode through CCD image. Incident light was not distributed well by pillar structures (Fig. 3b) because it remained confined in the central region of the light-trapping layer. Hence, the focus intensity was high in the middle of the pattern but weaker towards the edges, which indicates that the incoming light was not uniformly dispersed by each pattern. Lens arrays with rough surfaces (Fig. 3c) performed similarly to standard lens array patterns. Overall, these results show that each shape responds differently to incoming light. Therefore, more detailed analyses of the light distribution of each and how this affects the performance of DSSCs are required.

**Figure 4:**
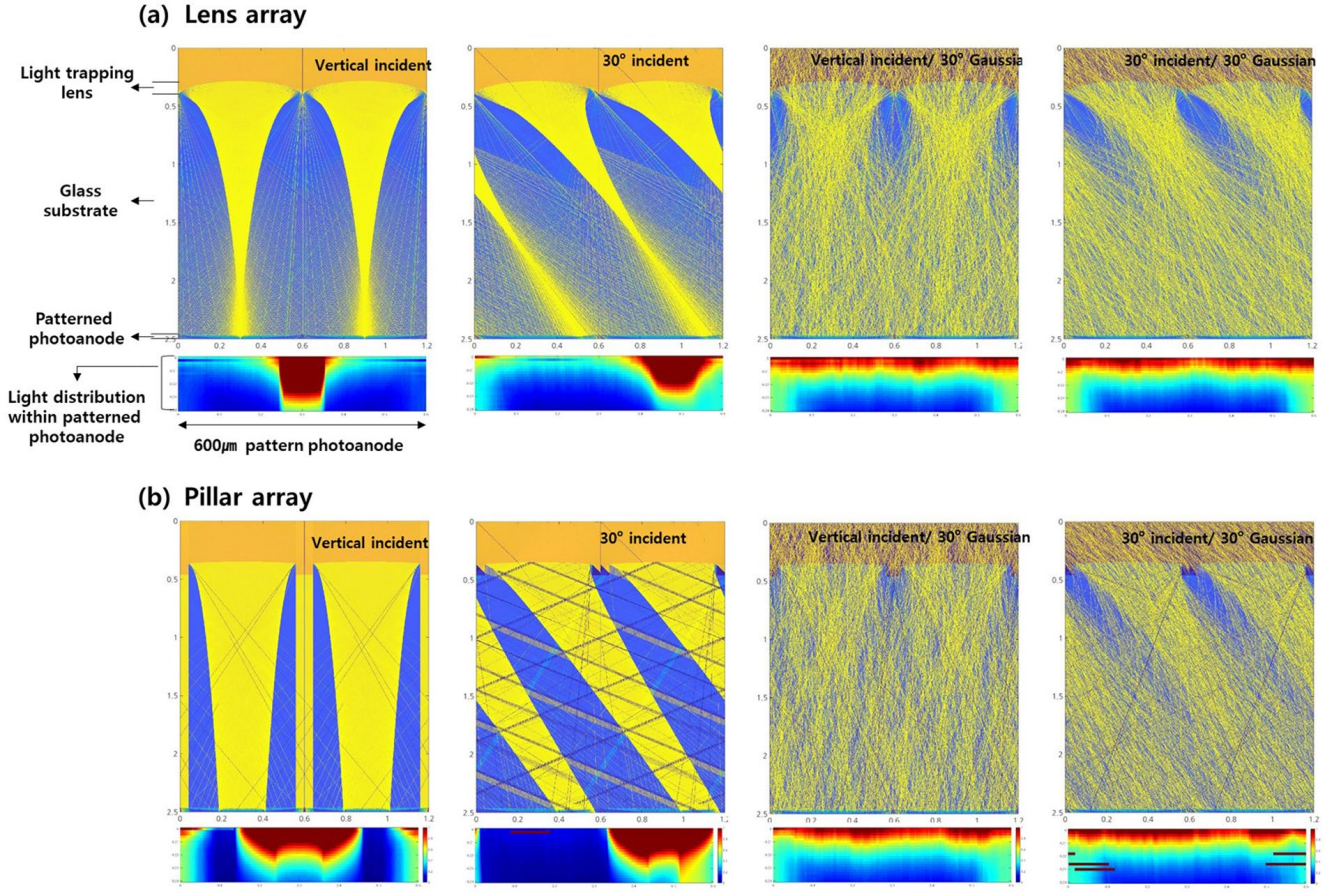
Light distribution and intensity within patterned photoanodes incorporating a (**a**) lens array and (**b**) pillar-shaped lens array, according to two-dimensional ray-tracing analyses. The labels ‘vertical incident’ and ‘30 degree incident’ indicate that the incident beam was vertical or at an angle of 30 degrees to the cell. The angle of incidence of each photon following a Gaussian distribution centered on the vertical and ranging up to 30 degrees is labeled ‘vertical incident/30 degree Gaussian’; that centered at 30 degrees with a range of 30 degrees is labeled ‘30 degree incident/30 degree Gaussian’.

Before incorporating these components into DSSCs, we investigated their light path modification behavior using a 2D ray-tracing method. We simulated a 600 µm patterned photoanode with a light-trapping layer incorporated into it. The results are shown in Fig. 4. We used two main structures, the lens array and the pillar array, in our calculations. We analyzed the scanning electron microscopy (SEM) images shown in Fig. 2 to measure their dimensions. The distribution of light in photoanodes of 35 µm thickness was initially evaluated for two incoming light conditions: a perfectly parallel vertically incident beam (‘vertical incident’) and at a 30° angle from the vector in the normal direction to the cell (‘30 degree incident’). These two conditions are generally assumed when designing the light-trapping components of solar cells, although the actual incoming light is not a perfectly aligned beam. Thus, to also take this into account, we considered two further cases: in the third condition, the angles of each incident photon were taken from a Gaussian distribution centered at the vertical and ranging up to 30° (‘vertical incident/30 degree Gaussian’); the fourth condition also assumed that the incident angles followed a Gaussian distribution with a range of 30° but were centered at 30 degrees (‘30 degree/30 degree Gaussian’). Cases using other Gaussian angles of incidence are shown in Fig. S5.

In the case of perfectly aligned vertical beams, the focal point of the lens array layer was located just above the photoanodes. Therefore, both focused light and light that deviated from the vertical was distributed through the photoanodes. We obtained similar results in the ‘30 degree incident’ case but the beam was focused at a different location. However, the incident light became homogeneously distributed within the photoanodes when the angles of incidence were sampled from Gaussian distributions, as shown in Fig. 4(a). Similar results were observed in the case of pillar structures but the light was focused on a location beyond the photoanodes; the distribution of the perfectly aligned beam did not deviate within the photoanodes. Therefore, the obliquely incident light was concentrated near the edges of photoanodes, where the charge generation was somewhat restricted by the lower thickness compared to the centers of the photoanodes. One interesting feature was exhibited in the case of the Gaussian angles of incidence. In the case of vertically incident Gaussian light, the lateral light distribution by the pillar-shaped array was more homogenous near the photoanodes, while the lens array layer homogeneously distributed 30° incident light more laterally. (Light distribution results at other angles of incidence are shown in Fig. S6) We suspect that this variation would have different effects on the efficiencies of DSSCs at different angles of incidence.

Also before observing the DSSCs performance with light-trapping layer depends on angle of incident, we have measured optical transmittance of PDMS film which is used for the light-trapping layer (Fig. S7). The transmittance of PDMS shows similar value without significant difference depends on oblique angle.

**Figure 5:**
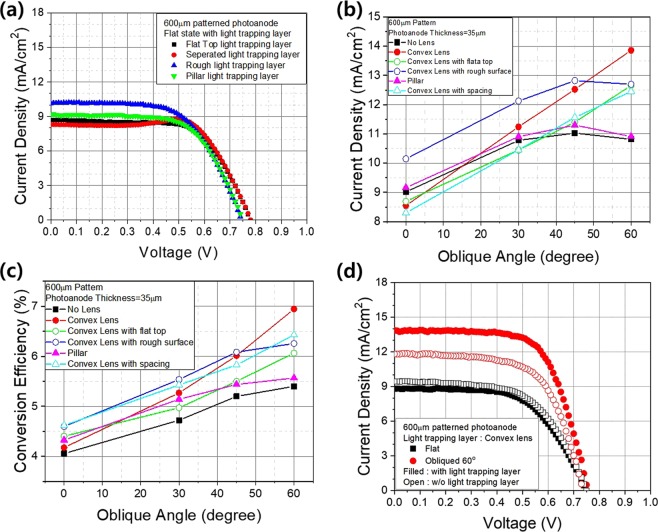
(**a**) Dependence of the relationship between the current density and voltage of 600 µm patterned photoanode DSSCs on the type of light-trapping layer under 1 sun AM1.5 conditions. (**b**) Current density and (**c**) dependence in (**a**) with vertically, 30°, 45°, and 60° obliquely angled incident light under 1 sun AM1.5 conditions. (**d**) Dependence with and without a light-trapping layer with vertically and 60° obliquely angled incident light under 1 sun AM1.5 conditions.

Based on the photoanode projection area with respect to the vertically and obliquely incident light, we determined that the different light-trapping layers yielded DSSCs with different current densities and energy-conversion efficiencies, as shown in Fig. 5. We obtained the best current density and efficiency values from vertically incident light with the lens array with rough surfaces. The current density obtained from the pillar shaped array was superior to that of DSSCs without a light-trapping layer (Fig. 5a,b). However, although the energy-conversion efficiency is not exclusively determined by the current density, it was improved by all the light-trapping layers (Fig. 5c). Therefore, the light-trapping layer plays a role in regulating the reactions at the interfaces, thus maintaining their light passing balance between individual components. Further studies are required to elucidate the cause of this effect. The results varied significantly as the angle of incidence increased. Both the current density and efficiency were enhanced to a great extent by the lens array layer. We obtained similar improvements from lenses with flat tops and rough surfaces. Although the shapes of the lenses were similar, the distribution of the light depended strongly on the morphology of the surface, particularly in the case of obliquely incident light. The efficiency of the conversion of obliquely incident light from an angle of 60° in DSSCs with lens array layers was almost 70% higher than that of DSSCs without light-trapping layers for vertically incident light, as shown in Fig. 5(d). These results confirm that the light-trapping layers deliver critical omnidirectional light-trapping capabilities, confirming their expected role in leaf anatomy, and that light-trapping performance varies according to their shape. To summarize these results, pillar-shaped arrays are recommended in the case of vertically incident light, whereas lens shapes are advantageous when light is obliquely incident and/or scattered. It may be possible to construct highly effective light-trapping layers by combining both shapes, as shown in Fig. 1(d), in which case we can expect to obtain high-performance DSSCs that are capable of converting light from any direction into energy.

**Figure 6:**
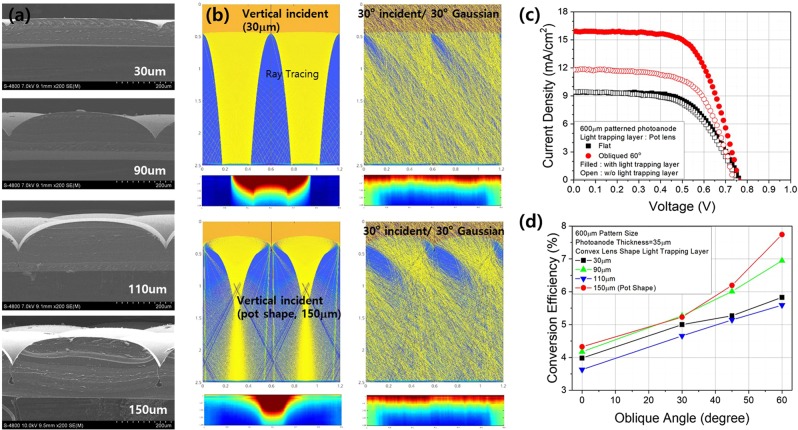
(**a**) Scanning electron microscopy (SEM) images of the dependence of the performance of the lens array light-trapping layer on the height of the lens. (**b**) Light distribution and intensity within the patterned photoanode using lenses of heights 30 µm (up) and 150 µm (down) incident vertically (left) on the cell and with each photon having a Gaussian angle of incidence with the Gaussian distribution centered at 30 degrees and with a range of 30 degrees (right). (**c**) Relationship between the current density and voltage of 600 µm patterned photoanode DSSCs with or without a pot-shaped lens array-based light-trapping layer in cases of vertically or 60° obliquely angled incident light under 1 sun AM1.5 conditions. (**d**) Dependence of the conversion efficiency of such DSSCs with lens array-bases layers on the height of lenses in cases of vertically and 30°, 45°, and 60° obliquely angled incident light under 1 sun AM1.5 conditions.

As mentioned previously, we can vary the light-trapping performance and distribution behavior of lenses of the same shape by making geometric adjustments. We investigated the effects of varying the height of the lens from 30 µm to 150 µm by controlling the depth of the etching process of the Si wafer used as the master mold, as shown in Fig. 6(a). This figure shows a very interesting feature for a lens height of 150 µm: pot-shaped lenses. However, the middle parts of each lens were connected, whereas the ends were separate. We predicted the light distribution behavior of these using ray-tracing simulations with variable lens heights. The results indicated that increasing the lens height focused the light closer to the surface of the DSSC. Beyond the focal point of both perfectly aligned and Gaussian distributed light, incident either vertically or obliquely, the light spread homogeneously into the patterned photoanodes (Fig. 6b). The dependency of the distribution of the light on the geometric properties of the light-trapping layer, such as the height of the lens, also affected the performance of the DSSCs (Fig. 6c,d). The pot-shaped lens array with lenses of 150 µm height exhibited the best conversion efficiencies for both vertically (4.33%) and obliquely incident light (7.74%).

Simply increasing the height of the lens did not induce higher conversion efficiencies, as evidenced by the fact that 90 µm lenses had higher conversion efficiencies than 110 µm lenses. Therefore, further research employing precise calculations of lens patterns with different shapes and dimensions is required. This will help us determine the best-performing light-trapping layer.

## Conclusion

We have proposed a new strategy for designing light-trapping layers, by selecting structures that mimic those of leaf epidermal cells. Based on this idea, we developed several novel light-trapping layers with various shapes and dimensions and confirmed that incorporating them into DSSCs improved their performance, particularly with respect to capturing omnidirectional light. However, this study only provides the initial proof-of-concept for improving the performance of DSSCs by mimicking structures with botanical origins. Much further research is required to address the questions raised by this study. In particular, we should investigate methods for analyzing plants from a structural perspective, particularly focusing on the precise characterization of the structures of leaves. This will enable us to invent novel analytical techniques that are better suited to our needs. It is also essential to understand the principles governing how the anatomical components that we are mimicking operate and interact with the environment. The promising results of this study potentially represent a great opportunity for improving PV devices, or even developing the next generation of such devices. We hope that our findings will serve as the first step in adapting the mechanisms evolved by plants and applying them to the development of maximally optimized solar cells.

## Experimental Details

### Calculations

The light distributions within the DSSCs were calculated by two-dimensional (2D) ray tracing for paraxial rays and rays with Gaussian initial incident directions centered at different angles. The calculations were performed using in-house code written in MATLAB®. For the calculations, the geometries were divided into 1 μm pixels and reorganized into the 2D refractive index map traversed by the rays. The rays propagated without changing direction until they reached an interface, indicated by the change in the refractive index, where they changed direction in accordance with Snell’s Law. The energy of the change in direction was determined by the Fresnel equation. Rays were simultaneously reflected from and refracted by the interface. We recorded and analyzed the passing traces of the traveling rays, including the incident rays and the rays generated by the reflection at the interface, from the initial position to the end of the map.

### Fabrication of light-trapping layers

The master mold had a silicon (Si) wafer substrate. For patterning, photoresist (PR, AZ 9260) layer was coated onto the Si wafer at 3500 rpm for 30 s to obtain a coating of thickness 7 µm utilizing spin coater (Midas System) then baking the wafer at 110 °C for 10 s. The photoresist was patterned by photolithography, exposed to ultraviolet (UV) radiation using a mask aligner (MA-6, Suss Microtec), then removed using developer (THMA 2.38%) so that we could engrave each of the shapes, sized 600 µm, of the hexagonal array. We etched the samples using a dry-etching process with a deep reactive ion etcher (ICP Etcher, SPTS) with cycling Bosch etching and isotropic etching. Etching process was carried out under condition that result in etching rate of approximately 10 µm per minute (Pressure 30 mTorr, Source power 2400 W, Bias power 130 W, Gas SF_6_/O_2_). Then the etched Si wafer was cleaned in preparation for the next step.

We fabricated light-trapping layers by coating polydimethysiloxane (PDMS, SR-580, Heesung STS) onto the master mold using a spin coater (VSF-150 MD) at 2000 rpm for 40 s and then baking it at 70 °C for 20 min. Then these were peeled off the master mold.

The etching process was commissioned by the National Institute for Nanomaterials Technology, Pohang University Science and Technology.

### DSSC cell preparation

F-doped SnO_2_ (FTO, sheet resistance 7Ω sq^−1^, Sigma Aldrich) glass was used as the electrode substrate. The FTO glass was rinsed with acetone, ethanol, and deionized water, subjected to sonication for 30 min, and then dried with nitrogen gas. A blocking layer (Solaronix) was deposited onto the FTO glass by automatic screen printing (AutoMax) then heat-treated at 530 °C for 3 h in air. We deposited 20 nm TiO_2_ nanoparticles (Solaronix) onto the blocking layer and the 1 cm^2^ activation area, which was treated in the same way for both the uniform and patterned electrodes; then this was heat-treated at 500 °C in air for 1 h. TiO_2_-deposited FTO glass was immersed in 0.3 mM ethanol solution (Sigma Aldrich) with N719 dye (Sigma Aldrich) at room temperature for 20 h. For the counter electrode, we applied the doctor blade method by masking the area on FTO glass using the 3 M tape then deposition platinum (Pt) paste (Solaronix) onto FTO glass, and then removing the masking tape. Then deposited Pt paste were heat-treated it at 450 °C in air for 30 min.

The semi-solid electrolyte was deposited on dye-loaded photoanode and counter electrode using the doctor blade method with 3 M masking tape. The semi-solid electrolyte was composed of 1.38 M 1-ethyl-3-methylimidazolium iodide (EMII, Sigma Aldrich), 0.07 M iodine (Sigma Aldrich), 0.13 M guanidine thiocyanate (Sigma Aldrich), 0.85 M 4-tert-butylpyridine (Sigma Aldrich), and 0.7 M lithium iodide (Sigma Aldrich) in succinonitrile (SN, Sigma Aldrich). The volume ratio of SN to EMII was 3:1. We have added Silica (SiO_2_) nano-power (50 nm, Amstec.) with weight ratio of SN to SiO_2_ nano-powder was 3.16:1. We assembled the two electrodes deposited onto the semi-solid electrode using a press machine, and then sealed them with ultraviolet-cured bond (Laser Bond USA). We constructed an array of four DSSCs connected in parallel using a flat flame, and used oblique flames at angles of 30°, 45°, and 60° for the sub-modules.

### Characterization

We evaluated the surfaces and cross-sections of the patterned electrodes and light-trapping layers and measured the thicknesses of the electrodes by analyzing images obtained by field-emission scanning electron microscope (FE-SEM, Hitachi S4800). For imaged of light trapping effect, we have conducted capturing CCD image utilizing CCD Camera (Mightex CCE-C013-U), Lens (AZURE-1214 mm) and light source (Magnum-F, LED source). The camera, light-trapping layer and light source were placed horizontally and then the image was obtained by adjusting focus of the lens (Fig. S4). For optical transmittance of PDMS film, we utilized UV-Vis –NIR Spectrophotometer (Cary 5000, Varian).

To assess the photovoltaic performance of the DSSCs, we first calibrated a solar simulator (Abet Technologies, model Sun 2000, 1000 W Xenon source, Keithley 2400 source meter) with a KG-3 filter and an NREL-certified reference cell, then operated it under 1 sun AM1.5 conditions.

## Supplementary information

Supplementary Information
