# Supplementary material for: Omnidirectional Light Capture by Solar Cells Mimicking the Structures of the Epidermal cells of Leaves
Source: Sci Rep. 2019 Sep 4;9:12773. doi: 10.1038/s41598-019-49046-8 (PMC6726600; doi:10.1038/s41598-019-49046-8)
Supplement: Supplementary file 1 — Supplementary Information [file 41598_2019_49046_MOESM1_ESM.docx]

**Supplementary Information**

**Omnidirectional Light Capture by Solar Cells Mimicking the Structures of the Epidermal cells of Leaves**

**Min Ju Yun^1^, Yeon Hyang Sim^1,2^, Seung I. Cha*^1,2^, Dong Y. Lee^1,2^**

**1. Energy Conversion Research Center, Creative and Fundamental Research Division,**

**Korea Electrotechnology Research Institute**

**2. Department of Electro-functionality Materials Engineering, University of Science and Technology**

*Correspondence to Dr. Seung I. Cha, Korea Electrotechnology Research Institute, Boolmosan-ro 10beon-gil, Seongsan-gu, Changwon 51543, Korea. E-mail: sicha@keri.re.kr; Tel: +82-55-280-1649


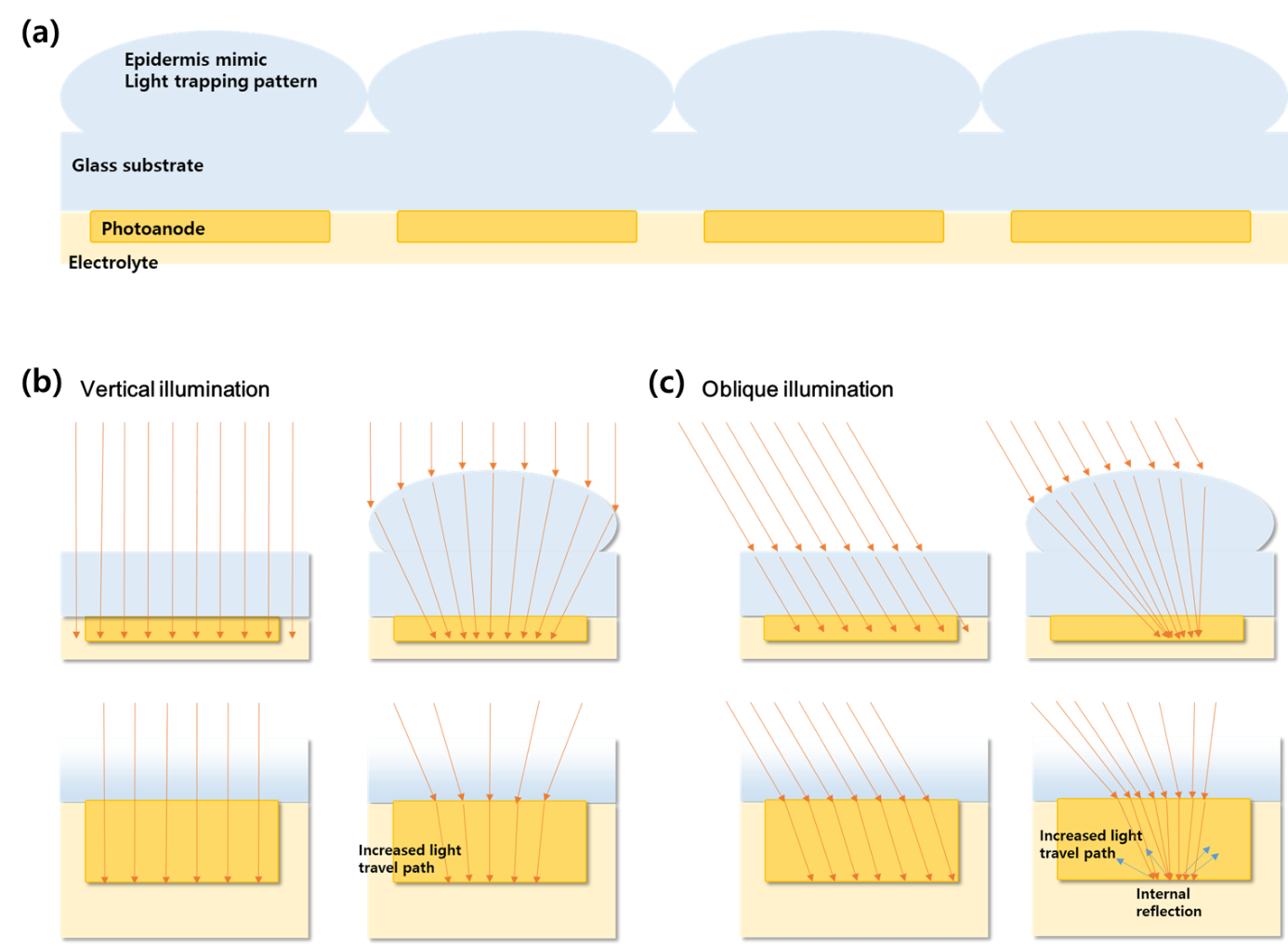


**Figure S1.** Illustration of (a) DSSC with light-trapping layer and patterned photoanode, incident light pass way at (b) vertical and (c) oblique illumination with and without light-trapping layer.


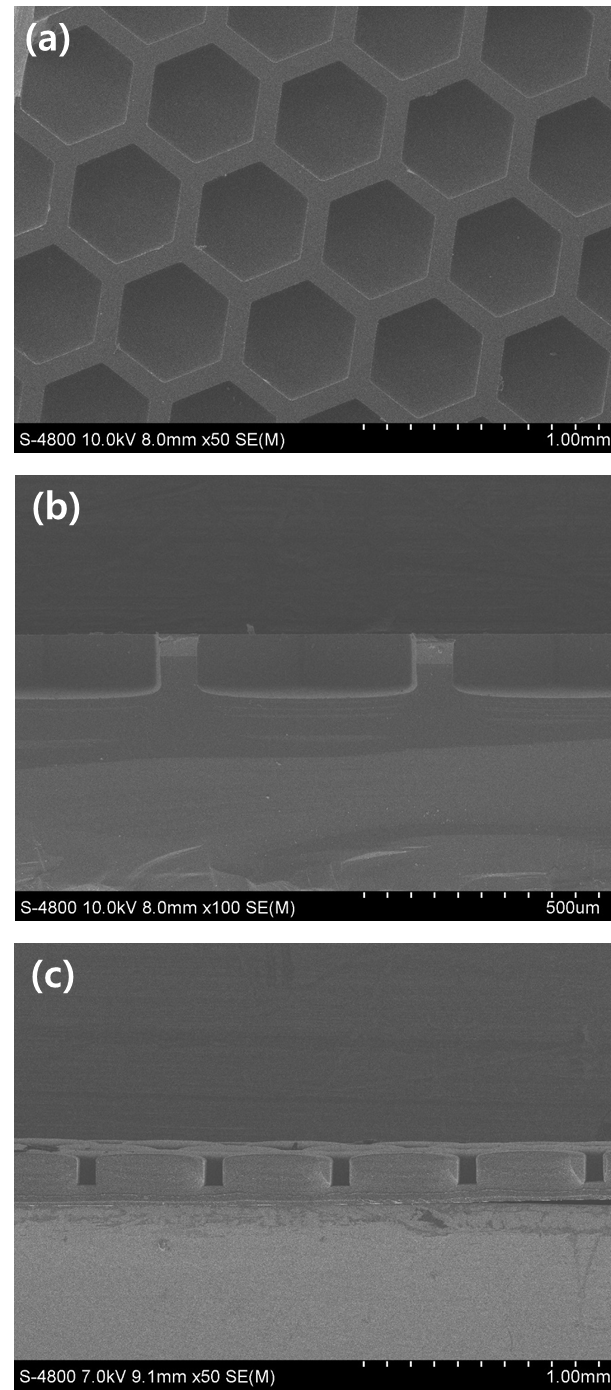


**Figure S2.** SEM images (a) plane and (b) cross section view of etched Si wafer as a master mold and (c) light trapping layer of pillar shaped lens array.


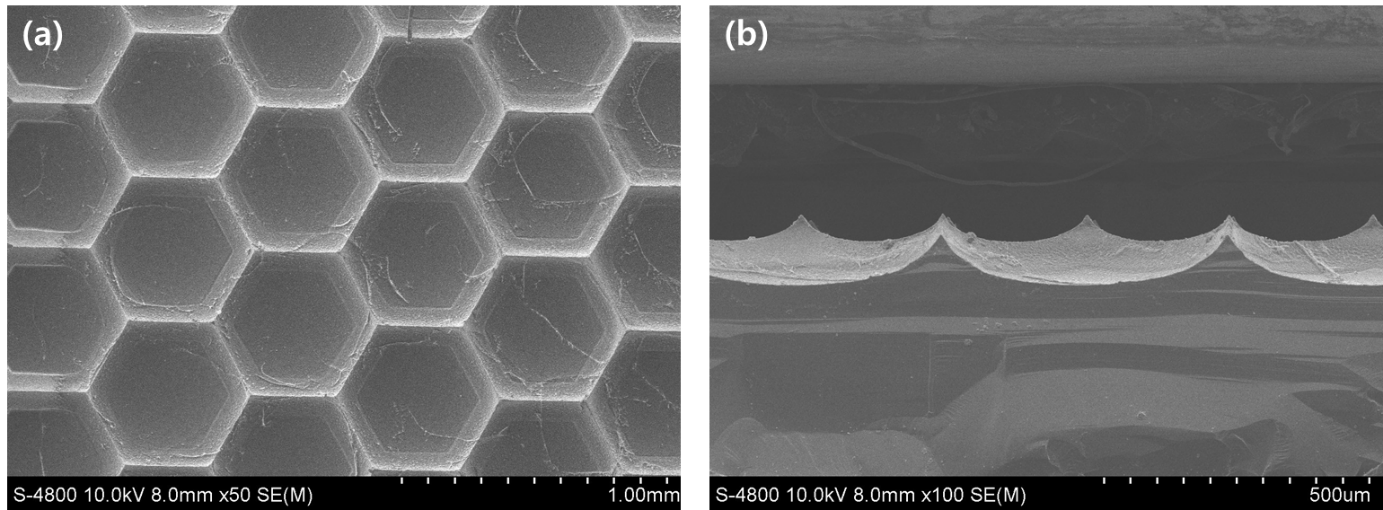


**Figure S3.** SEM images (a) plane and (b) cross section view of etched Si wafer as a master mold of lens array with rough surface.

**
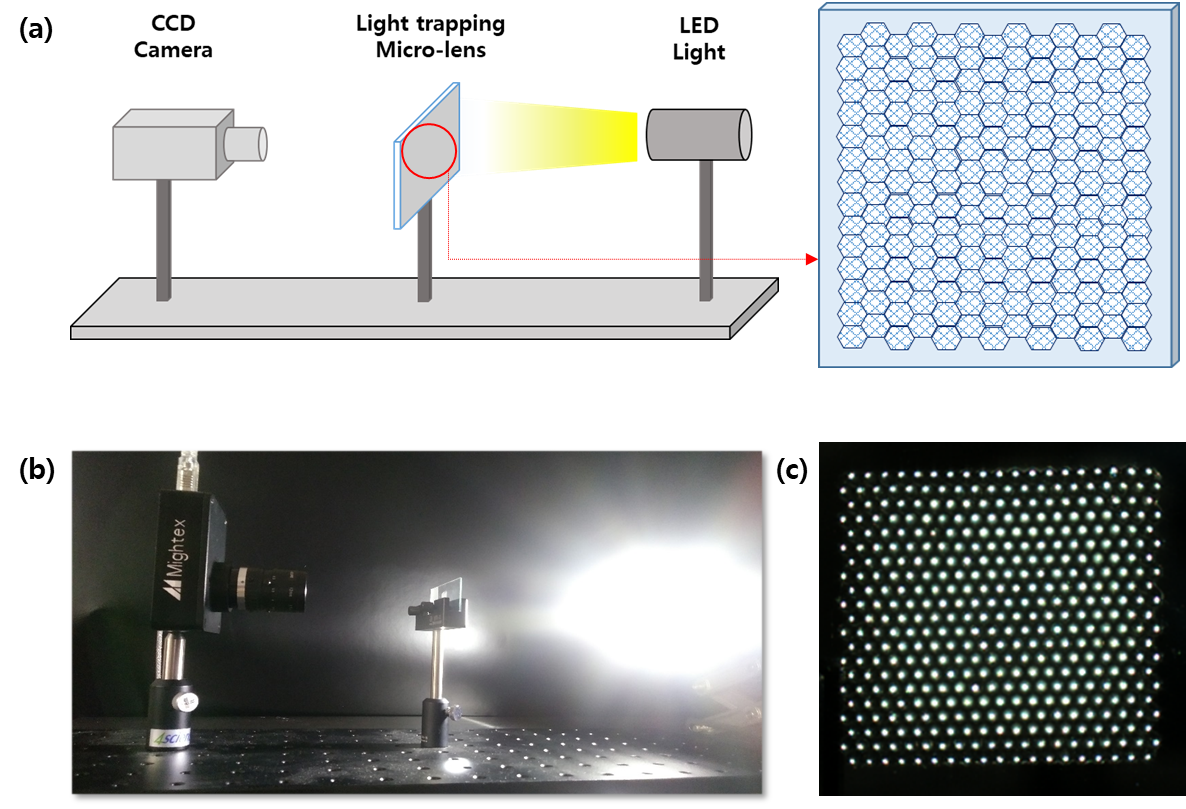
**

**Figure S4.** (a) Schematic illustration of setting up with CCD camera, light trapping lens and light source and (b) photograph of actual measurement and (c) CCD image of focused incident light through light-trapping lens.

**
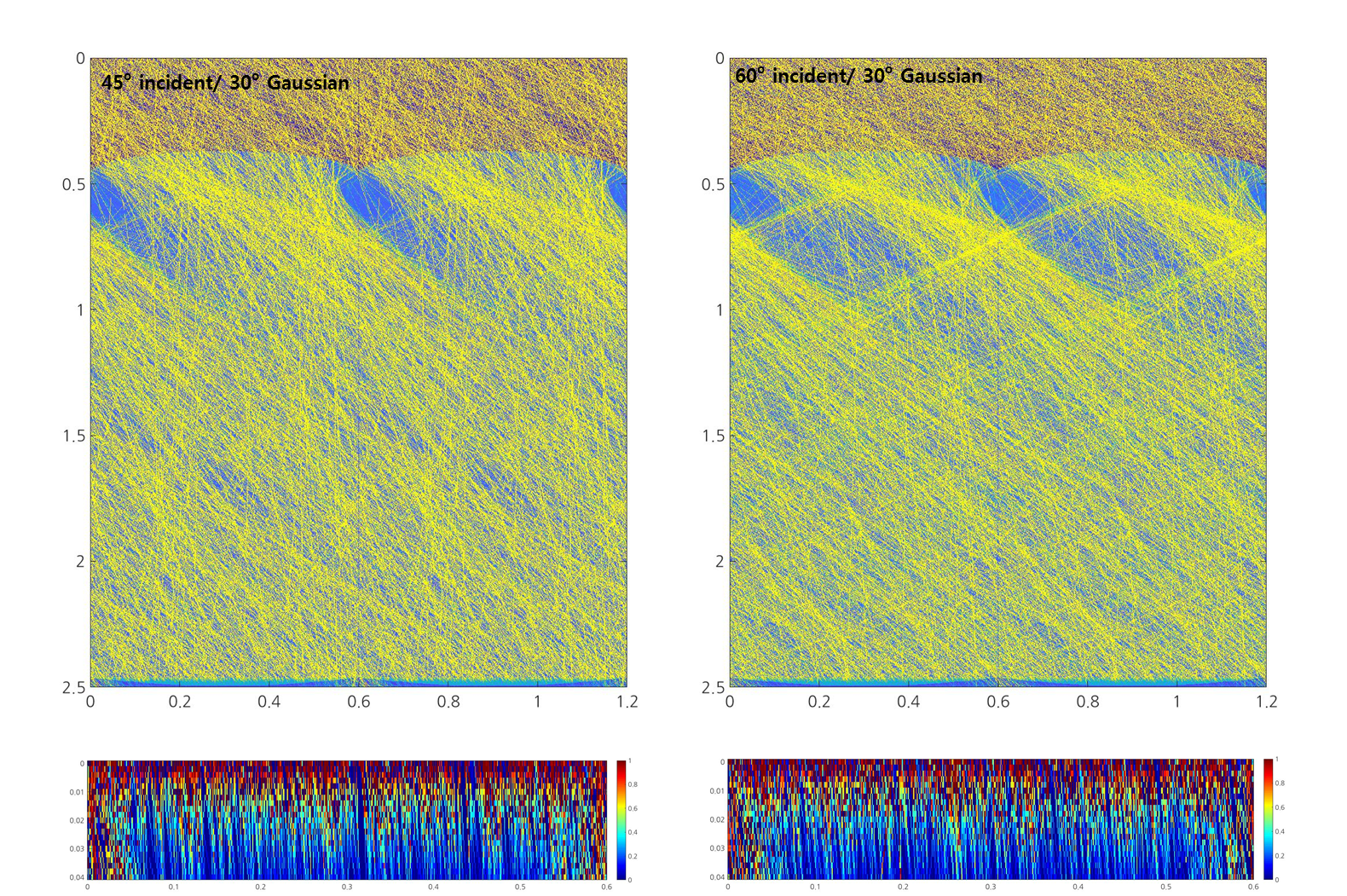
**

**Figure S5.** Light distribution and intensity within patterned photoanode introducing lens array by 2D ray tracing analysis. Incident angle of each photon has Gaussian distribution centered at 45 degree incident and ranged with 30 degree marked ‘45 degree incident/30 degree Gaussian’, and 60 degree incident and ranged with 30 degree marked ‘45 degree incident/30 degree Gaussian’.

**
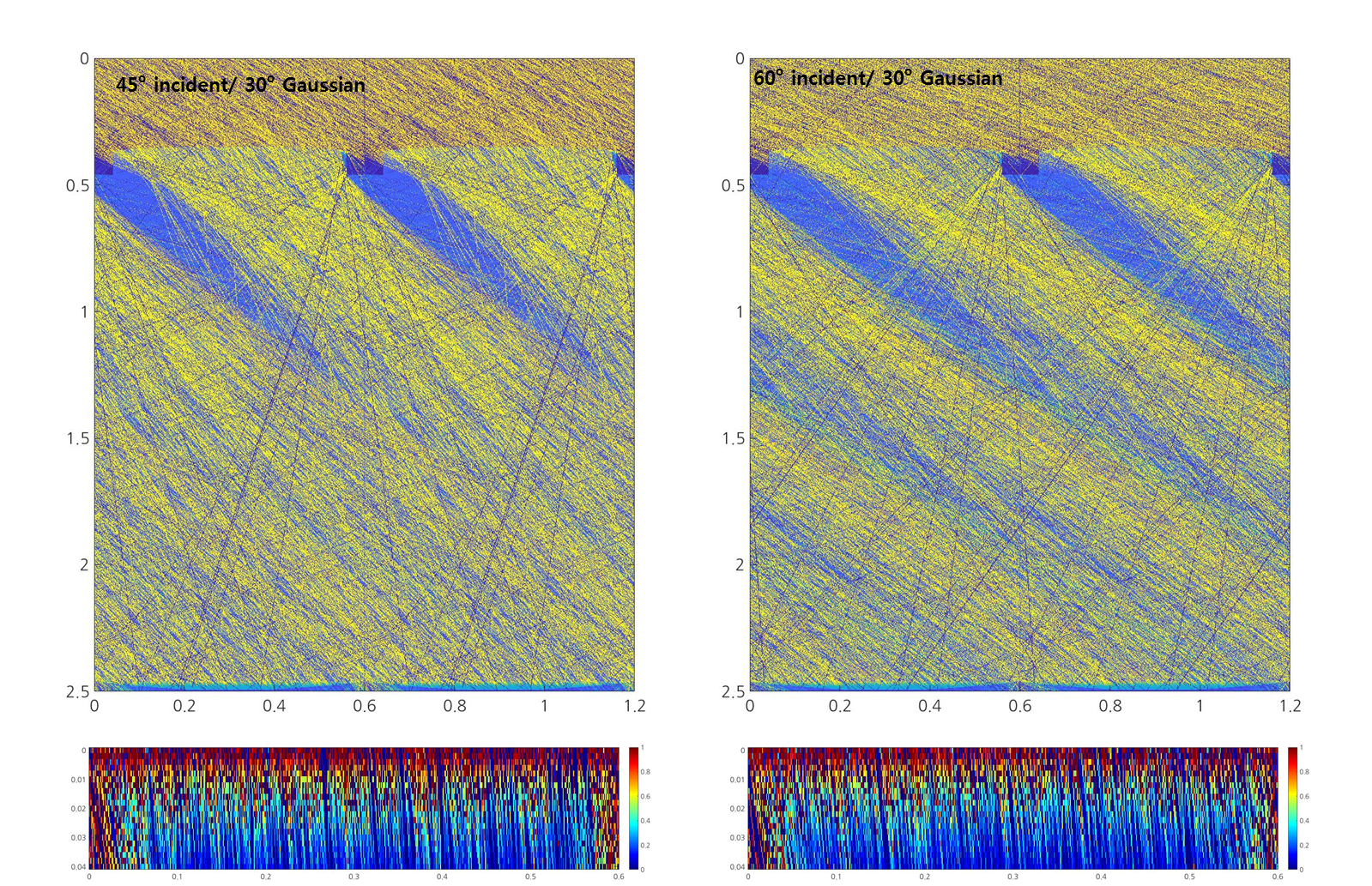
**

**Figure S6.** Light distribution and intensity within patterned photoanode introducing pillar shaped lens array by 2D ray tracing analysis. Incident angle of each photon has Gaussian distribution centered at 45 degree incident and ranged with 30 degree marked ‘45 degree incident/30 degree Gaussian’, and 60 degree incident and ranged with 30 degree marked ‘45 degree incident/30 degree Gaussian’.

**Figure S7.** Transmittance of PDMS film depends on oblique angle 0 to 30 degree.
